# Natural steroid-based cationic copolymers cholesterol/diosgenin-*r*-PDMAEMAs and their pDNA nanoplexes: impact of steroid structures and hydrophobic/hydrophilic ratios on pDNA delivery†

**DOI:** 10.1039/d1ra00223f

**Published:** 2021-06-01

**Authors:** Zhao Wang, Jingjing Sun, Mingrui Li, Ting Luo, Yulin Shen, Amin Cao, Ruilong Sheng

**Affiliations:** Department of Radiology, Shanghai Tenth People's Hospital, School of Medicine, Tongji University Shanghai 200072 China; School of Material Engineering, Jinling Institute of Technology Nanjing 211169 China; CAS Key Laboratory of Synthetic and Self-assembly Chemistry for Organic Functional Molecules, Shanghai Institute of Organic Chemistry, Chinese Academy of Sciences 345 Lingling Road Shanghai 200032 China; CQM-Centro de Quimica da Madeira, Universidade da Madeira Campus da Penteada Funchal Madeira 9000-390 Portugal ruilong.sheng@staff.uma.pt

## Abstract

Using natural-based lipids to construct biocompatible, controllable and efficient nanocarriers and elucidating their structure–function relationships, was regarded as an important area for creating sustainable biomaterials. Herein, we utilized two natural steroids: cholesterol and diosgenin (bearing different hydrophobic tails) as the building blocks, to synthesize a series of natural steroid-based cationic random copolymers PMA6Chol-*r*-PDMAEMA and PMA6Dios-*r*-PDMAEMA *via* RAFT polymerization. The results demonstrated that the steroid-*r*-PDMAEMA copolymers could efficiently bind pDNA (N/P < 3.0) and then form near-spherical shape (142–449 nm) and positively-charged (+11.5 to +19.6 mV) nanoparticles. The *in vitro* cytotoxicity and gene transfection efficiency greatly depend on the steroid hydrophobic tail structures and steroid/PDMAEMA block ratios. Optimum transfection efficiency of the (Chol-P1/pDNA and Dios-P3/pDNA) nanoplexes could reach to 18.1–31.2% of the PEI-25K/pDNA complex. Moreover, all of the steroid-*r*-PDMAEMA/Cy3-pDNA nanoplexes have an obvious “lysosome localization” effect, indicating the steroid structures do not remarkably influence the intracellular localization behaviors of these nanoplexes.

## Abstract 2

Using natural-based lipids to construct biocompatible, controllable and efficient nanocarriers and elucidating their structure–function relationships, was regarded as an important area for creating sustainable biomaterials.

## Graphical abstract

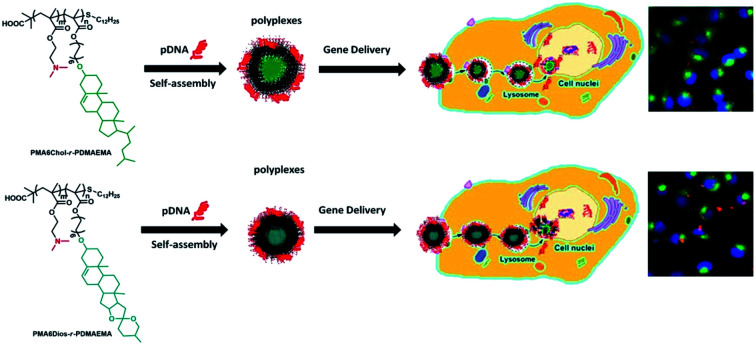

## 1. Introduction

Cationic polymers as non-viral gene carriers have been intensively studied in recent decades, due to their controllable structure and function, excellent siRNA/pDNA condensing capability, as well as low immunogenicity.^1,2^ Although many cationic polymer carriers have been developed to realize efficient gene transfection, some defects, such as low solution stability, poor serum compatibility, high cytotoxicity and low cellular uptake, have still largely restricted their practical applications.^3^ To overcome the defects, it was disclosed that conjugation/modification of cationic polymers on their backbones/side chains with biocompatible building blocks: polyethylene glycol (PEG),^4–6^ carbohydrates,^7,8^ zwitterionic molecules^9^ and natural products,^10^ was an efficient way to reduce cytotoxicity and improve bioavailability. Meanwhile, researchers made great efforts to elucidate the structure–function relationships of cationic polymers so as to obtain the optimal balance between gene transfection efficiency and cytotoxicity.^11–14^

Introducing hydrophobic moieties into cationic polymers could improve plasmid (pDNA) binding/loading affinity and nanoplex stability by facilitating the formation of “hydrophobic core–hydrophilic shell” structure, they could also increase membrane fusion and cellular uptake, which gave rise to enhanced gene transfection.^15^ Hydrophobic moieties, including polyesters,^16–20^ poly(meth)acrylate^21^ and natural lipids^22,23^ were incorporated into cationic polymers to prepare stable nanoparticles, microcapsules or liposomes. Xu *et al.*^24^ revealed that phenylalanine could enhance the gene transfection efficacy of poly(amidoamine)s (PAMAM) in SMMC-7721 and COS-7 cells. Lee *et al.*^25,26^ found that dexamethasone-conjugated polyethylenimine (PEI)/PAMAM have higher gene delivery efficiency than their unmodified counterparts in acute lung injury (ALI) animal models. Dong *et al.*^18–20,27–31^ developed a series of polyester-based cationic polymers with enhanced pDNA (or siRNA) transfection efficacy. Moreover, changing hydrophobic core in nanoparticles (NPs) could affect the delivery efficiency of NPs/gene nanoplexes by changing their endocytic pathways and intracellular siRNA release behavior. In a previous work, we disclosed that introducing proper length of hydrophobic poly(l-lactide) into poly(l-lysine) could increase pDNA binding, nanoplex stability, and improve gene transfection capability through an efficient caveolae-mediated endocytosis pathway.^32,33^ In some cases, introducing natural lipids, such as cholesterol, phospholipid and vitamin E, could effectively decrease cytotoxicity and improve gene transfection efficiency.^34–37^ Eliyahu *et al.*^38^ modified dextran-spermine conjugated cationic polymer with natural oleic acid, which showed improved transfection capability in NIH-3T cells. Likewise, Neamnark *et al.*^39^ found aliphatic lipid modification of PEI-25K would greatly enhance its transfection efficiency. Nevertheless, up to date, the relationship between hydrophobic (lipid) moiety/block ratios of cationic gene carriers and their apparent cytotoxicity and gene transfection efficiency, is still not fully addressed.

Natural steroids (especially cholesterol) were recently utilized as renewable, biocompatible and hydrophobic lipid moieties to construct efficient gene carriers.^40^ Bhattacharya *et al.*^41^ disclosed that some cholesterol-modified oligoethylenimine (Cho-OEI) polymers possessed higher gene transfection efficiency than the “gold standard” bPEI-25K. Davis *et al.*^42,43^ prepared cholesterol-containing random cationic polymer gene (pDNA) carriers from cholesteryl methacrylate (CMA) and (dimethylamino)ethyl methacrylate (DMAEMA) monomers, the optimum transfection ratio was achieved on 2% cholesterol molar ratio (N/P = 20). Dufès *et al.*^44^ designed disulfide-linked cholesterol-PEGylated diaminobutyric polypropylenimine G3 dendrimers, the introduction of cholesterol could enhance the stability of nanovesicles and promote drug/gene co-delivery efficiency. Lee *et al.*^45^ synthesized cholesterol-conjugated polyamidoamine (PAM-Chol) for combined delivery of anti-inflammatory gene and drug into the lungs by inhalation, which showed higher transfection efficiency than lipofectamine 2000 and bPEI-25K in L2 lung epithelial cells. Moreover, it was revealed that introducing cholesterol to cationic gene carriers could offer them some specific biological manners *e.g.* Zhang *et al.*^46^ discovered that some siRNA-loaded cholesterol-grafted bioreducible poly(amidoamine)s (rPAA-Ch) nanoparticles got into cell through caveolae-dependent endocytosis pathway and localized in lysosomes. In an earlier work, we synthesized a series of cationic cholesterol-disulfide (CHOSS) lipids with low cytotoxicity and high gene transfection efficiency in COS-7 cells, their pDNA nanoplexes showed an interesting “perinucleic localization” effect.^47^ However, apart from cholesterol, other steroid-based cationic gene carriers were rarely prepared and studied, the structural dependence of their self-assembly properties, cytotoxicity, gene transfection capability, endocytosis and intracellular trafficking/localization, were still remain obscure.^48^ Noteworthy, most of the steroidal compounds have similar four-ring core skeletons but different hydrophobic tails, which made them good model molecules for further investigation of the structure–function relationships.

PDMAEMA has been considered as an excellent gene carrier since its high transfection efficiency with acceptable cytotoxicity and buffering capacity (known as “proton sponge effect”) for endosomal escape. PDMAEMA and its copolymers could be easily synthesized by atom transfer radical polymerization (ATRP) or reversible addition fragmentation transfer (RAFT) polymerization method with controllable molecular weights and architectures (*e.g.* random and block), providing an efficient molecular engineering approach for structure–function relationship study.^49–51^

Based on the above background, to explore the effects of steroid hydrophobic moieties on gene transfection, in this work, we prepared series of new steroid-based cationic random copolymers (steroid-*r*-PDMAEMA), including PMA6Chol-*r*-PDMAEMA (Chol-P1, P2, P3) and PMA6Dios-*r*-PDMAEMA (Dios-P1, P2, P3), *via* RAFT polymerization of cholesterol-based monomer MA6Chol (or diosgenin-based monomer MA6Dios) with the DMAEMA monomer. Their structures were characterized by nuclear magnetic resonance (NMR) and gel permeation chromatography (GPC). pDNA binding affinities of the as-synthesized steroid-*r*-PDMAEMA copolymers were determined by agarose gel retardation assay, the average particle sizes, zeta potentials and morphologies of steroid-*r*-PDMAEMA/pDNA nanoplexes were analyzed by dynamic laser scattering (DLS) and transmission electron microscope (TEM), respectively. *In vitro* cytotoxicity of the steroid-*r*-PDMAEMA copolymers was evaluated by CCK-8 assay and pDNA transfection efficiency was measured by luciferase activity assay in H1299 lung cancer cell line. Moreover, intracellular localization of the PMA6Chol_4_-*r*-PDMAEMA_190_ (Chol-P1)/Cy3-pDNA and PMA6Dios_18_-*r*-PDMAEMA_180_ (Dios-P3)/Cy3-pDNA nanoplexes was observed by fluorescence microscopy.

## 2. Experimental

### 2.1 Materials

Cholesterol (97.0%) and diosgenin (98.0%) were purchased from Acros Organics. *P*-Tosylchloride and 1,6-hexanediol (98.0%) were purchased from TCI, Japan. Methylacrylic acid (98.0%) was purchased from Lingfeng Chemical Co Ltd. Shanghai. Dicyclohexylcarbodiimide (DCC, 98.0%) and 4-dimethylaminopyridine (DMAP, 99.0%) were purchased from GL Biochem Ltd. Azodiisobutyronitrile (AIBN, 98.0%) was purchased from Aladdin, Shanghai and re-crystallized twice in methanol before use. Branched structural poly(ethyleneimine) (bPEI-25K, *M*_w_ = 25 000) was purchased from Sigma-Aldrich. Dimethylaminoethylmethacrylate (DMAEMA, 99.0%) was purchased from Aladdin, Shanghai, and purified with basic aluminum oxide to remove the inhibitor. RAFT chain transfer agent (CTA) 2-(dodecylthiocarbonothioylthio)-2-methylpropionic acid (DDMAT) was synthesized referred to the literature.^52^ The organic solvent toluene was freshly distilled before use. All other solvents and chemicals were purchased from commercial suppliers and used as-received. Luciferase assay and bicinchoninic acid (BCA) protein quantitation kits (Cat #PW0104) were supplied by Promega (USA) and Biomiga (USA), respectively. DAPI (Cat #70217321) was purchased from Roche, Switzerland. Label IT-Cy3 Nucleic acid labeling kit (Cat #MIR7010) was purchased from Mirus Bio Corporation (USA). Cell counting kit-8 (CCK-8, Cat #MG6432) and LysoTracker Green (Cat #MF8124G) were purchased from MesGen Biotech. (China). PCMV-Luc plasmid (pDNA) was kindly gifted by Prof. Yuhong Xu of school of pharmacy of Shanghai Jiaotong University. RPMI1640 medium was purchased from Hangzhou Genom Co., Ltd. Fetal bovine serum (FBS) was purchased from GIBCO (Cat #10099-141, Life Technology). Human lung cancer cell line H1299 was generously gifted by Dr Bo Wan of Fudan University (Shanghai, China).

### 2.2 Preparation of the steroid-*r*-PDMAEMA cationic polymers

The steroid-*r*-PDMAEMA cationic polymers were prepared by controllable RAFT radical polymerization. Firstly, the cholesterol-based MA6Chol and diosgenin-based MA6Dios monomers were synthesized *via* three-step process (S1, ESI†). Then the as-synthesized MA6Chol/MA6Dios monomers and RAFT chain transfer agent (CTA) were RAFT-polymerized in the presence of initiator AIBN to prepare the resulting final product steroid-*r*-PDMAEMA cationic polymers: PMA6Chol-*r*-PDMAEMA and PMA6Dios-*r*-PDMAEMA as light yellow powder. The details of the polymerization were depicted in S2, ESI.†

#### PMA6Chol-*r*-PDMAEMA

^1^H NMR (CDCl_3_, *δ* in ppm): 5.31–5.35 (C=C*H* of cholesterol), 4.08–4.19 (COOC*H*_2_), 3.43–3.49 (C*H*_2_OChol), 3.12–3.14 (OC*H*R of cholesterol), 2.53–2.63 (C*H*_2_N of DMAEMA), 2.23–2.39 ((C*H*_3_)_2_N of DMAEMA).

#### PMA6Dios-*r*-PDMAEMA

^1^H NMR (CDCl_3_, *δ* in ppm): 5.32–5.35 (C=C*H* of diosgenin), 4.08–4.19 (COOC*H*_2_), 3.41–3.48 (C*H*_2_ODios), 3.11–3.15 (OC*H*R of diosgenin), 2.53–2.63 (C*H*_2_N of DMAEMA), 2.23–2.39 ((C*H*_3_)_2_N of DMAEMA).

### 2.3 Structural characterization of the steroid-*r*-PDMAEMA cationic polymers

^1^H NMR spectra were recorded on a Bruker Avance-300 NMR spectrometer at ambient temperature, operating at 300.0 MHz for proton (^1^H) nuclei. Molecular weights (*M*_n_, *M*_w_) and their distribution (*M*_w_/*M*_n_) were characterized on a PerkinElmer 200 gel permeation chromatography (GPC) equipped with a refractive index detector (RI), and double TSKgel multipore H_XL_-M columns and a guard column (TOSO, Japan) were employed with THF as the eluent at a flowing rate of 1.0 mL min^−1^. Polystyrene standards with narrow molecular weight distribution (Polymer laboratories, UK) were used to generate the calibration curve. The molecular weights and distribution (*M*_w_/*M*_n_) were calculated with attached PE GPC software.

### 2.4 Agarose gel retardation assay

Before the agarose gel assay, the PMA6Chol-*r*-PDMAEMA/pDNA and PMA6Dios-*r*-PDMAEMA/pDNA nanoplexes were prepared by mixing plasmid DNA (1.0 μg) with a predetermined amount of PMA6Chol-*r*-PDMAEMA or PMA6Dios-*r*-PDMAEMA cationic copolymers to a total volume of 30 μL. After incubation at 37 °C for 20 min, the polyplex solution was loaded onto a 1.0% agarose gel containing 0.5 μg mL^−1^ ethidium bromide (EB). Then gel electrophoresis was set up in 1× TAE running buffer at 100 mV and kept for 40 min. Finally, the retardation of DNA migration was observed and recorded on a UVP benchtop 2UV transilluminator instrument.

### 2.5 Dynamic light scattering (DLS)

Average particle size and zeta potential of the as-prepared PMA6Chol-*r*-PDMAEMA/pDNA and PMA6Dios-*r*-PDMAEMA/pDNA nanoplexes under various N/P ratios were analyzed on a Malvern Zetasizer Nano ZS90 DLS instrument at 25 °C with incident laser beams at *λ* = 633 nm and a scattering angle of 90°.

### 2.6 Transmission electronic microscopy (TEM)

The as-prepared PMA6Chol-*r*-PDMAEMA/pDNA and PMA6Dios-*r*-PDMAEMA/pDNA nanoplexes (N/P = 3) solution was dropped onto a 300-mesh carbon-coated copper grid, and excess fluid was removed with filter paper, then air-dried at room temperature. The morphologies of the nanoplexes were observed by TEM (JEOL-1230, JEOL Co. Ltd, Japan) with an acceleration voltage of 80 kV.

### 2.7 Cytotoxicity of the steroid-*r*-PDMAEMA by cell counting kit-8 (CCK-8) assay

Cytotoxicity of the PMA6Chol-*r*-PDMAEMA and PMA6Dios-*r*-PDMAEMA cationic copolymers was examined by CCK-8 assay. First, H1299 cells were seeded in 24-well microplates with 500 μL RPMI 1640 medium containing 10% FBS at a density of 5 × 10^5^ cells per well and incubated for 24 h. Then the predetermined amount of PMA6Chol-*r*-PDMAEMA (or PMA6Dios-*r*-PDMAEMA) cationic copolymer solution was added and incubated with human lung cancer H1299 cells (4 × 10^4^ per well) at 37 °C for 24 h, and CCK-8 solution (10 μL per well) was added and incubated for 4 h in 96-well plates. The absorption of CCK-8 was measured at 450 nm with a background correction of 620 nm using ELISA reader (Bio-Rad). Relative cell viability (%) was calculated as (OD_sample_ − OD_0_)/(OD_control_ − OD_0_) × 100%. All data was presented as mean value with standard deviation of three experiments done in triplicate (*n* = 3).

### 2.8 *In vitro* transfection assay

H1299 cells were first inoculated in 24-well microplates with 500 μL RPMI 1640 medium containing 10% FBS at a density of 4 × 10^5^ cells per well and cultivated for 24 h. Then, the medium in microplates were replaced with serum-free RPMI 1640 medium. The as-prepared PMA6Chol-*r*-DMAEMA/pDNA and PMA6Dios-*r*-PDMAEMA/pDNA nanoplexes at various N/P ratios (1.0 μg pDNA per well) was added and kept incubation at 37 °C for 4 h. Thereafter, the medium was replaced with 500 μL of fresh RPMI 1640 medium with 10% FBS and further incubated at 37 °C for 24 h. Luciferase gene transfection assays were conducted according to the protocol of Promega luciferase assay system, and total protein contents were measured using BCA protein quantification kit. Gene transfection efficiency was calculated as relative light units per milligram of luciferase protein (RLU per mg). Commercially available bPEI-25K was conducted in the same way as the control. All transfection experiments were performed in triplicate (*n* = 3).

### 2.9 Intracellular localization of the polyplexes

Firstly, Chol-P1/Cy3-pDNA and Dios-P3/Cy3-pDNA nanoplexes were previously prepared by mixing the polymers with Cy3-pDNA (N/P = 3) and incubating at 37 °C for 30 min. Intracellular localization of the PMA6Chol-*r*-PDMAEMA/Cy3-pDNA and PMA6Dios-*r*-PDMAEMA/Cy3-pDNA polyplexes was observed by fluorescence microscope. H1299 cells were seeded into 6-well plate (2 × 10^5^ cells per well) and cultivated at 37 °C overnight in RPMI 1640 medium supplemented with 10% FBS and 1% penicillin–streptomycin. Subsequently, the cells were separately transfected with the as-prepared Chol-P1/Cy3-pDNA or Dios-P3/Cy3-pDNA polyplexes. After 4 h incubation, the cells were washed with PBS for three times, then fixed with 4% paraformaldehyde in 0.12 M phosphate buffer, pH 7.2, at 37 °C for 30 min and washed three times in PBS buffer. Afterward, DAPI (100 ng mL^−1^, for cell nuclei staining) and Lysotracker (1 μg mL^−1^, for lysosome staining) were added and further incubated for 15 min, then washed with PBS. The fluorescence images were observed and recorded on a Nikon Ti–S invert fluorescence microscopy.

## 3. Results and discussion

### 3.1 Preparation of the steroid-*r*-PDMAEMA cationic copolymers PMA6Chol-*r*-PDMAEMA and PMA6Dios-*r*-PDMAEMA

**Scheme 1:**
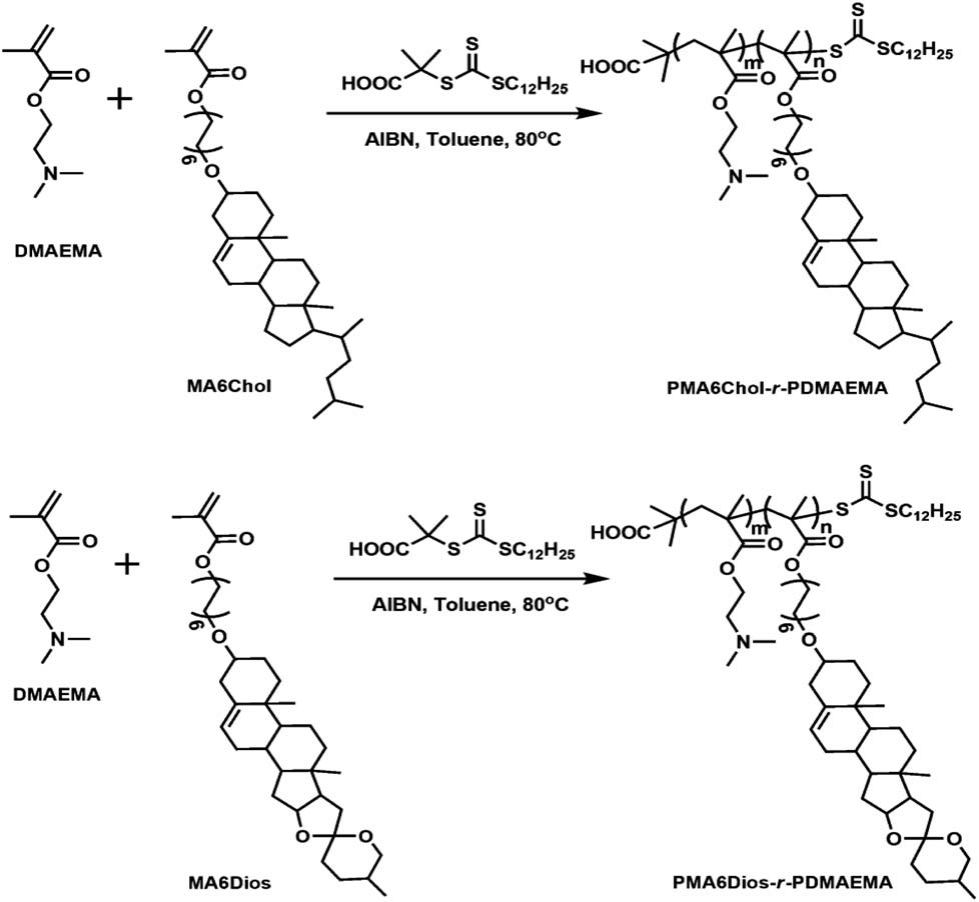
Synthetic routes of the PMA6Chol-*r*-PDMAEMA (Chol-P) and PMA6Dios-*r*-PDMAEMA (Dios-P) random copolymers, the polymerization reaction time were set as 12 h, *n* and *m* represent the average degrees of polymerization for the hydrophobic (MA6Chol or MA6Dios) blocks and hydrophilic blocks (DMAEMA), respectively.

**Fig. 1:**
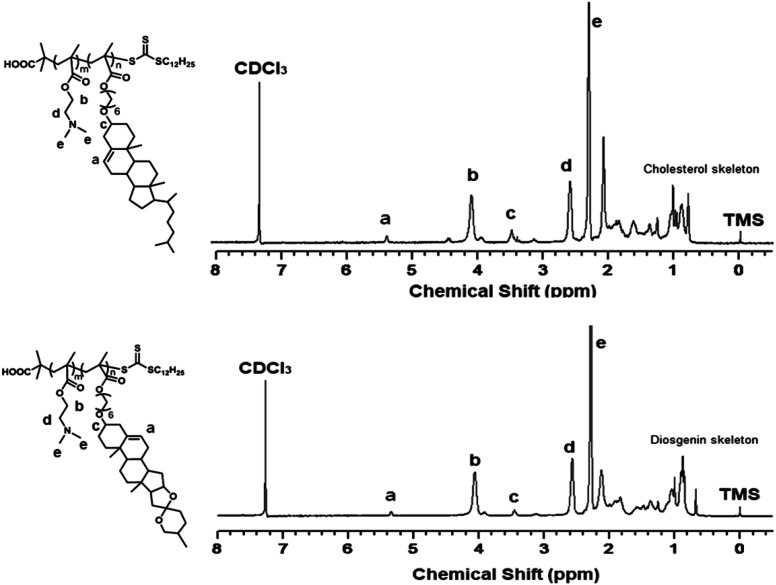
^1^H NMR spectrum for the PMA6Chol_17_-*r*-PDMAEMA_189_ (Chol-P3, top) and PMA6Dios_18_-*r*-PDMAEMA_180_ (Dios-P3, bottom) random copolymers in CDCl_3_.

First, the cholesterol-based monomer MA6Chol and diosgenin-based monomer MA6Dios were synthesized *via* a three-step organic synthetic strategy. Details of the polymer preparation process and ^1^H NMR spectra were shown in the ESI (S1, Scheme S1(a) and (b) and Fig. S1(a) and (b)†). Then, the MA6Chol and MA6Dios monomers were copolymerized with DMAEMA *via* reversible addition fragmentation transfer (RAFT) polymerization method, respectively, with the DDMAT as the chain transfer agent (CTA) and AIBN as the initiator (Scheme 1). DDMAT have hydrophobic alkyl chain (log *P* ∼ 6.9) and polar carboxyl group, which makes it highly compatible to the steroid-bearing monomer and DMAEMA monomer. Moreover, compared to aromatic ring-bearing CTA,^8^ the electron-donating property of alkyl chain on DDMAT could bring good reactivity to thioester free radicals. By regulating the monomer feeding ratios, a series of PMA6Chol-*r*-PDMAEMA (Chol-P1, P2 and P3) and PMA6Dios-*r*-PDMAEMA (Dios-P1, P2 and P3) random copolymers were prepared, with the steroid-bearing hydrophobic weight ratios of 7–27 wt%. Notably, the PMA6Chol-*r*-PDMAEMA and PMA6Dios-*r*-PDMAEMA random copolymers showed very close average polymerization degrees of each blocks and hydrophilic/hydrophobic weight ratios, which benefits for elucidating their structure–function relationships. Table 1 summarizes synthetic results for the PMA6Chol-*r*-PDMAEMA and PMA6Dios-*r*-PDMAEMA copolymer series. Fig. 1 shows the typical ^1^H NMR spectrum for as-resulted Chol-P3 and Dios-P3 random copolymers, in which ^1^H nuclei resonance signals a (5.2 ppm) and c (3.4 ppm) could be assigned to the double bonds (C=CH) and OC*H*R groups of steroid cores, and the proton signals b (4.1 ppm), d (2.6 ppm) and e (2.3 ppm) could be assigned to the DMAEMA moieties/units, respectively. The assigned ^1^H NMR resonance signals and high monomer conversions as well as low *M*_w_/*M*_n_ values indicated the successful preparation of the steroid-*r*-PDMAEMA copolymers with controlled structures by one-step RAFT polymerization approach.

**Table 1:** Characteristics of the synthesized PMA6Chol-*r*-PDMAEMA (Chol-P) and PMA6Dios-*r*-PDMAEMA (Dios-P) random copolymers^a^

Sample	DP_MA6Chol/MA6Dios_ (*n*)^a^	DP_DMAEMA_ (*m*)^a^	*M*_n,NMR_ (kg mol^−1^)	*M*_n,GPC_^b^ (kg mol^−1^)	PDI^b^	Molar ratio of MA6Chol/MA6Dios (%)	Hydrophilic/hydrophobic ratio (wt%)^c^
Chol-P1	4	190	3.25	2.92	1.50	2	93/7
Chol-P2	8	192	3.50	3.86	1.51	4	87/13
Chol-P3	17	189	3.95	4.30	1.38	8	76/24
Dios-P1	4	198	3.38	2.60	1.52	2	93/7
Dios-P2	9	193	3.59	2.66	1.51	4	85/15
Dios-P3	18	180	3.91	2.80	1.49	9	73/27

a Notes: ^a^DP_MA6Chol/MA6Dios_ (*n*) and DP_DMAEMA_ (*m*) represent the average degrees of polymerization for the hydrophobic blocks (MA6Chol or MA6Dios) and hydrophilic blocks (DMAEMA), respectively, evaluated on co-monomer conversions by ^1^H NMR. ^b^Number-average molar mass (*M*_n_) and polydispersity index (PDI, *M*_w_/*M*_n_) were determined by GPC with commercial polystyrene (PS) standard calibration. ^c^Calculated by the *M*_n,NMR_.

### 3.2 pDNA binding affinity of the PMA6Chol-*r*-PDMAEMA and PMA6Dios-*r*-PDMAEMA random copolymers

**Fig. 2:**
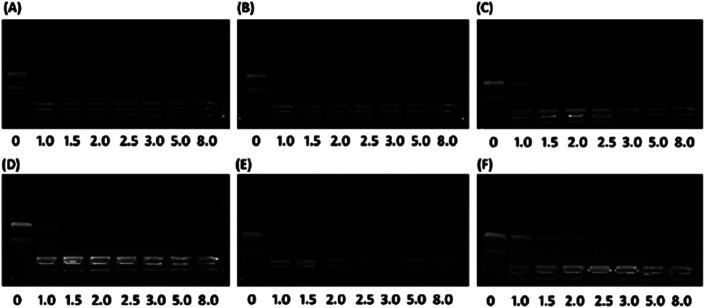
Agarose gel retardant assay of plasmid DNA affinity under various N/P charge ratios (0, 1.0, 1.5, 2.0, 2.5, 3.0, 5.0, 8.0) for the (A) Chol-P1/pDNA; (B) Chol-P2/pDNA; (C) Chol-P3/pDNA; (D) Dios-P1/pDNA; (E) Dios-P2/pDNA; (F) Dios-P3/pDNA.

To investigate the effect of hydrophobic steroid moieties and hydrophilic/hydrophobic ratios on gene binding/loading efficiency, we examined the pDNA binding affinity of PMA6Chol-*r*-PDMAEMA and PMA6Dios-*r*-PDMAEMA random cationic copolymers, which bearing various amount of hydrophobic cholesterol and diosgenin moieties/blocks but almost equal amount of DMAEMA cationic moieties/blocks, by agarose gel retardation assay. As shown in Fig. 2, all the PMA6Chol-*r*-PDMAEMA and PMA6Dios-*r*-PDMAEMA random copolymers could effectively bind and condense pDNA to completely retard its movement across the agarose gel at low N/P ratio of 2.5, indicating the strong capability for the prepared copolymers to condense pDNA into polyplexes. It is worth noting that the pDNA binding affinity could be achieved on the PMA6Chol-*r*-PDMAEMA and PMA6Dios-*r*-PDMAEMA cationic random copolymers with low hydrophobic ratios (cholesterol-containing blocks: 4, 8/per polymer; diosgenin-containing blocks: 4, 9/per polymer) at an N/P ratio of 1.0–1.5, while lower pDNA binding affinity (N/P ratio of 2.5) was observed on Chol-P3 and Dios-P3 (cholesterol-containing blocks: 17/per polymer; diosgenin-containing blocks: 18/per polymer) copolymers. The results illustrated that the pDNA binding affinity decreased along with the increasing of steroid ratio, which may be attributed to the steric hindrance effect of the large-sized steroid moieties. Moreover, mesogen properties of the steroid moieties may lead to the formation of self-organized micro-liquid crystal aggregation statement of the cationic copolymers, which further result in the formation of rigid and large-sized nanoplex aggregates and gave rise to lower pDNA binding affinities. Additionally, the pDNA binding affinity of cholesterol-based polymers seems slightly higher than that of diosgenin-based polymers, indicating the pDNA binding affinity did not only depend on the number of hydrophobic moieties, but also on the type of steroid architectures.

### 3.3 Particle size and zeta potential of the PMA6Chol-*r*-PDMAEMA/pDNA and PMA6Dios-*r*-PDMAEMA/pDNA nanoplexes measured by DLS

**Fig. 3:**
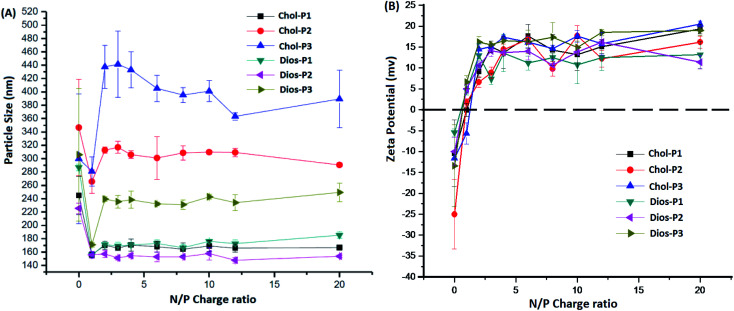
Average particle sizes (A) and zeta potentials (B) of the PMA6Chol-*r*-PDMAEMA/pDNA and PMA6Dios-*r*-PDMAEMA/pDNA nanoplexes under various N/P charge ratios by DLS.

It is known that average particle size and surface charge were important factors for the cytotoxicity, cellular uptake and gene transfection efficiency of the polyplexes.^31,32^ We measured the average particle size and zeta potential of the PMA6Chol-*r*-PDMAEMA/pDNA and PMA6Dios-*r*-PDMAEMA/pDNA nanoplexes by DLS with a fixed pDNA amount of 4.0 μg mL^−1^. As shown in Fig. 3(A), the average particle size decreased rapidly with the increasing of the ratio of the steroid-*r*-PDMAEMA copolymers when N/P ratio was lower than 2.0, indicating the efficient pDNA condensation effect.^8^ Then, the size of the nanoplexes increased with the increasing addition of cationic polymers and reached a platform at the N/P ratio of 3.0–4.0. Moreover, the particle size increased along with the increasing of hydrophobic cholesterol/diosgenin weight ratio, due to the enhanced aggregation induced by the increasing of hydrophobic moieties. Notably, the size of the cholesterol-containing PMA6Chol-*r*-PDMAEMA/pDNA polyplexes were larger than that of their diosgenin counterparts, indicating that the particle size depended on the type of the steroid hydrophobes, which might further influence their intracellular uptake and gene transfection properties. On the other hand, zeta potentials of the PMA6Chol-*r*-PDMAEMA/pDNA and PMA6Dios-*r*-PDMAEMA/pDNA were converted from negative to positive at the N/P ratio around 1.0–1.5, and then rapidly increased and maintained at +11.5 to +19.6 mV. The results indicated that the positive surface charge of the polyplexes was suitable for their intracellular uptake.

### 3.4 Morphology of the PMA6Chol-*r*-PDMAEMA/pDNA and PMA6Dios-*r*-PDMAEMA/pDNA polyplexes observed by TEM

**Fig. 4:**
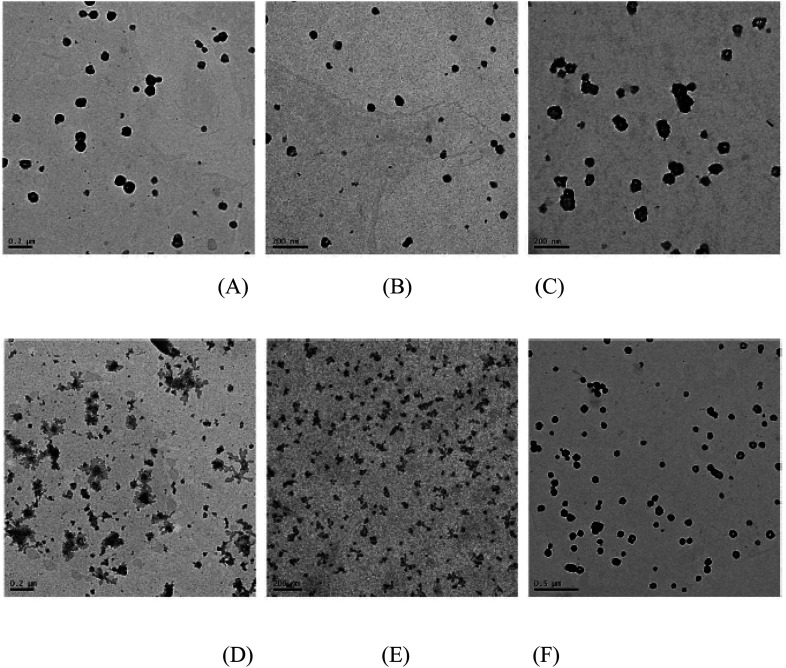
TEM images of the steroid-*r*-PDMAEMA/pDNA nanoplexes under N/P ratio of 3.0 for the (A) Chol-P1/pDNA; (B) Chol-P2/pDNA; (C) Chol-P3/pDNA; (D) Dios-P1/pDNA; (E) Dios-P2/pDNA; (F) Dios-P3/pDNA.

To further study the aggregation properties of the polyplexes, we observed the morphology of the PMA6Chol-*r*-PDMAEMA/pDNA and PMA6Dios-*r*-PDMAEMA/pDNA nanoplexes under the N/P ratio of 3.0 by TEM. As shown in Fig. 4(A, B, D and E), at low hydrophobic ratios (cholesterol/diosgenin-containing blocks: 4, 8 or 9/per polymer), it could be observed that the PMA6Chol-*r*-PDMAEMA/pDNA nanoplexes were near spherical-shaped nanoparticles with the size of 50–150 nm. Besides, PMA6Dios-*r*-PDMAEMA/pDNA were loose-structured nanoparticles with smaller size of 25–100 nm. At high hydrophobic ratios (17 or 18/per polymer, Fig. 4(C and F)), condensed aggregate structures were observed on both of the cholesterol/diosgenin-containing nanoplexes. The trend of the nanoplex particle size measured by TEM was in accordance with the DLS results, moreover, the comparatively smaller particle size observed by TEM could be attributed to the shrinkage of the hydrated nanoplexes during the sample preparation prior to TEM observation.^48^

### 3.5 *In vitro* cytotoxicity of the PMA6Chol-*r*-PDMAEMA and PMA6Dios-*r*-PDMAEMA random cationic copolymers evaluated by CCK-8 assay

**Fig. 5:**
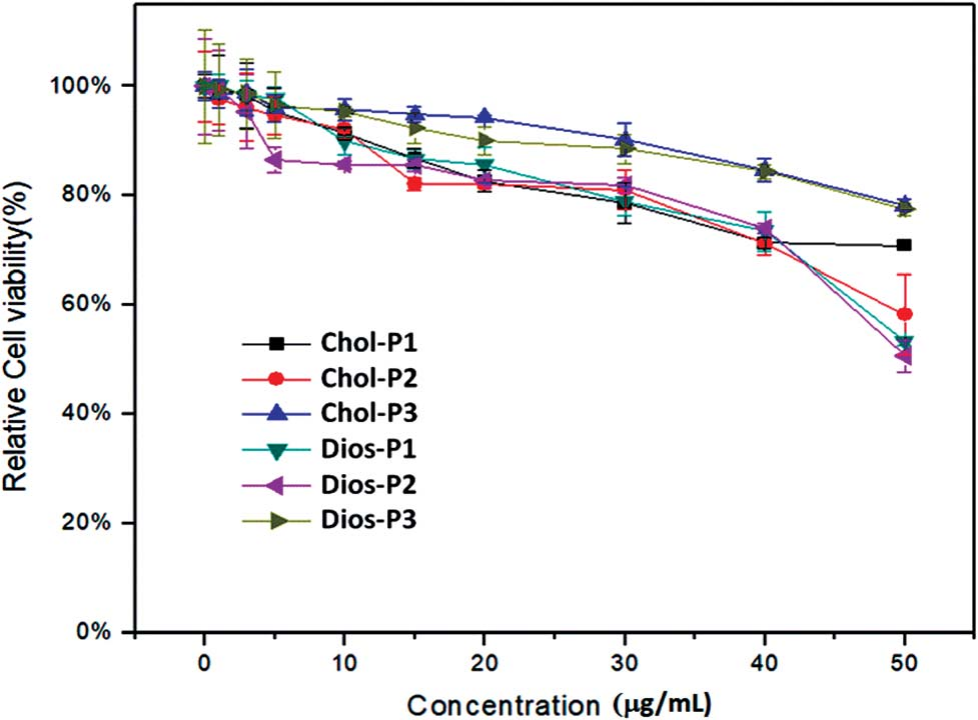
CCK-8 cytotoxicity assays for the PMA6Chol-*r*-PDMAEMA (Chol-P1, P2, P3) and PMA6Dios-*r*-PDMAEMA (Dios-P1, P2, P3) random cationic copolymers in H1299 cell line.

For safe gene transfection, the cytotoxicity of the cationic polymer was regarded as an essential factor.^31^ The *in vitro* cytotoxicity of PMA6Chol-*r*-PDMAEMA and PMA6Dios-*r*-PDMAEMA cationic copolymers were evaluated by CCK-8 assay in human lung cancer (H1299) cell line, the cell viability of free H1299 cells were set as 100%. As shown in Fig. 5, for the cationic copolymers with lower hydrophobic ratios (Chol-P1, Chol-P2, Dios-P1, Dios-P2), the H1299 cell viability decreased to 60% and below after incubation with 50 μg mL^−1^ each polymer, while with the increasing of hydrophobic ratio (Chol-P3 and Dios-P3, cholesterol/diosgenin-containing blocks: 18–19/per polymer), the H1299 cell viability still maintained around 80%. This may due to the formation of comparatively large-sized cationic polymer nanoparticle that diminished their electrostatic interactions with the negatively-charged plasma membrane. Similarly, Bhattacharya^41^*et al.* found that cholesterol-modified PEI have much lower cytotoxicity than unmodified PEI. Neamnark^39^*et al.* revealed that the cytotoxicity greatly relied on the structure of hydrophobic moieties, hydrophobic/hydrophilic proportion, as well as the hydrophobic modification ratio.^15^ Moreover, the results indicated that appropriate introducing hydrophobic steroid moieties to the cationic copolymers could further reduce the cytotoxicity, which offered the possibility for safe gene transfection.

### 3.6 Luciferase gene transfection efficiency of the PMA6Chol-*r*-PDMAEMA and PMA6Dios-*r*-PDMAEMA cationic random copolymers

**Fig. 6:**
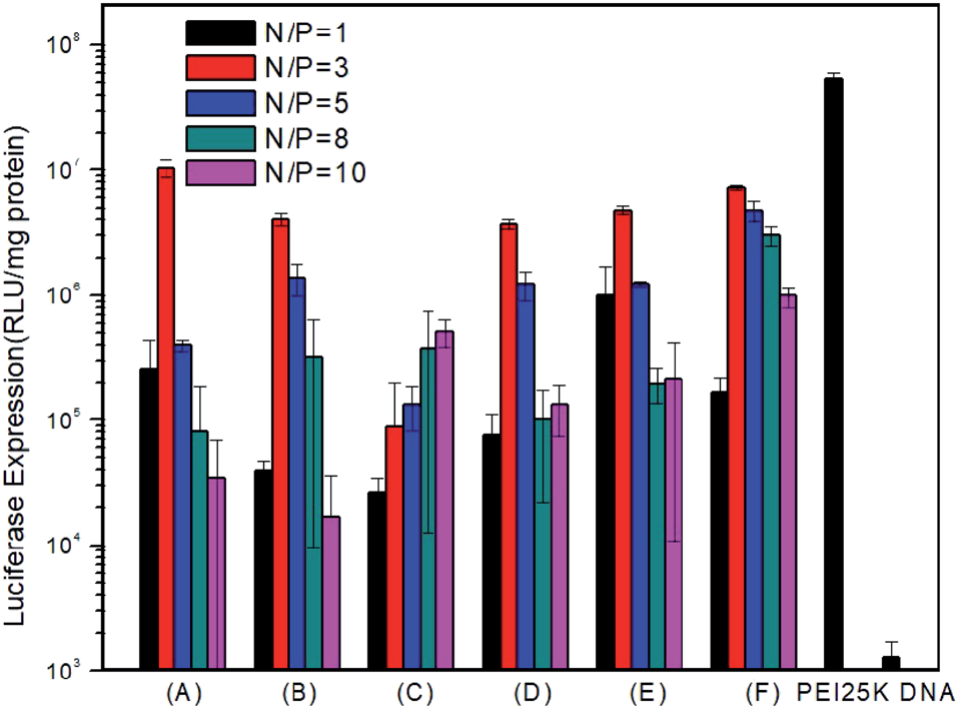
Luciferase gene transfection assay of H1299 cells transfected with PMA6Chol-*r*-PDMAEMA/pDNA: (A) Chol-P1 (B) Chol-P2 (C) Chol-P3 and PMA6Dios-*r*-PDMAEMA/pDNA: (D) Dios-P1 (E) Dios-P2 (F) Dios-P3 nanoplexes under various N/P charge ratios.

To examine *in vitro* gene transfection capability of the PMA6chol-*r*-PDMAEMA and PMA6Dios-*r*-PDMAEMA cationic copolymers, luciferase gene expression assay was conducted in H1299 cells. As shown in Fig. 6, gene transfection efficiency of the steroid-*r*-PDMAEMA random cationic polymers was found greatly depend on the hydrophobic/hydrophilic ratios and steroid hydrophobic tail structures. Among the copolymers, Chol-P1 and Dios-P3 showed maximum/optimum luciferase gene transfection capability at the N/P charge ratio of 3.0, and the optimum transfection efficiency could reach to 18.1–31.2% of the efficiency of commercially available “gold standard” PEI-25K.^48^ While higher hydrophobic cholesterol-containing Chol-P3 exhibit lower transfection capability, and the transfection efficiency enhanced with the increasing of N/P charge ratio within the range of measurement. The results were contrary to the trend of the size change of polyplexes. From this point of view, we can speculate that the low transfection efficiency of Chol-P3 may due to the large polyplex that hampered the intracellular uptake. On the contrary, Dios-P1, Dios-P2 and Dios-P3 showed increased transfection efficiency along with the increasing of diosgenin ratio, which may be attributed to their increasingly condensed micellar structures.

### 3.7 Intracellular localization of the PMA6Chol-*r*-PDMAEMA/Cy3-pDNA and PMA6Dios-*r*-PDMAEMA/Cy3-pDNA polyplexes

**Fig. 7:**
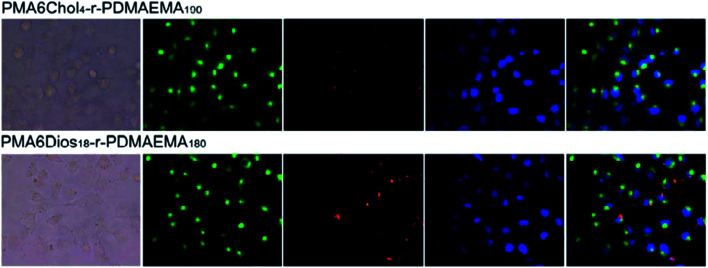
Fluorescence microscopic images (400×) of the localization of Cy3-labeled pDNA using PMA6Chol_4_-*r*-PDMAEMA_190_ (Chol-P1) and PMA6Chol_4_-*r*-PDMAEMA_180_ (Dios-P3) as the carriers in H1299 cells recorded after 4 h gene transfection (green: Lysotracker labeled lysosomes; red: Cy3 labeled pDNA; blue: DAPI stained cell nuclei).

To further understand the intracellular fate of the pDNA nanoplexes of PMA6Chol-*r*-PDMAEMA and PMA6Dios-*r*-PDMAEMA copolymers in H1299 cells, the optimized Chol-P1/Cy3-pDNA and Dios-P3/Cy3-pDNA nanoplexes were accordingly prepared, which then incubated with H1299 cells for 4 h and observed under fluorescence microscope. The cell nuclei and endosome/lysosome were separately stained with DAPI (blue) and Lysotracker (green), respectively. As shown in Fig. 7, the red fluorescence spotted inside the cells, indicating the successful and efficient cellular internalization of the Chol-P1/Cy3-pDNA and Dios-P3/Cy3-pDNA nanoplexes. It could be seen that some of the red fluorescent spots co-localized with green fluorescent dye Lysotracker, indicating that lysosome localization effect was involved in the cellular trafficking process of the Chol-P1/Cy3-pDNA and Dios-P3/Cy3-pDNA nanoplexes. Whereas a large portion of red fluorescent spots were not overlapped/co-localized with the Lysotracker, indicating that the Chol-P1/Cy3-pDNA and Dios-P3/Cy3-pDNA nanoplexes might possess “lysosome escape” effects, which might be attributed to the fast protonation of cationic PDMAEMA blocks inside lysosome. Besides, a small portion of red fluorescent spots were localized inside or around cell nuclei. It has been recently reported that some steroid-containing cationic gene carriers and their pDNA lipoplexes inclined to be taken up *via* lipid-raft or caveolae pathway,^48^ then the lipoplexes were future transported into endosome–lysosome, finally localized inside cell nuclei. Thus, in this work, we can suppose that the Chol-P1/Cy3-pDNA and Dios-P3/Cy3-pDNA might be internalized into H1299 cells through the gateway of “lipid-raft/caveolae-mediated endocytosis–endosome–lysosome–cell nuclei”. Further investigation on the endocytosis pathway of the PMA6Chol-*r*-PDMAEMA and PMA6Dios-*r*-PDMAEMA nanoplexes was carried out in our lab.

## 4. Conclusion

In summary, by using natural steroids (cholesterol and diosgenin) as the sustainable molecular resources, we synthesized a series of steroid-*r*-PDMAEMA cationic random polymers denoted as PMA6Chol-*r*-PDMAEMA (Chol-P1, P2, P3) and PMA6Dios-*r*-PDMAEMA (Dios-P1, P2, P3) *via* controllable RAFT polymerization. The steroid-*r*-PDMAEMA random cationic polymers could efficiently bind pDNA (N/P < 3.0) and subsequently form nanoparticles with near-spherical morphology (142–449 nm) and positive surface charge (+11.5 to +19.6 mV) in aqueous solution, and the particle size were depended on the hydrophobic/hydrophilic ratios and steroid structures. The cytotoxicity of the synthesized steroid-*r*-PDMAEMA random cationic polymers was relatively low, and increasing the hydrophobic/hydrophilic ratios could further diminish the cytotoxicity. The gene transfection efficiency of the steroid-*r*-PDMAEMA cationic random polymers was found greatly dependent on the hydrophobic/hydrophilic ratios and steroid structures, and the optimum transfection efficiency could reach to 18.1–31.2% of the efficiency of commercially available PEI-25K. The steroid-*r*-PDMAEMA/Cy3-pDNA polyplexes showed a well-known “lysosome localization” effect, which may facilitate their gene transfection process. Moreover, all of the steroid-*r*-PDMAEMA/Cy3-pDNA nanoplexes have obvious “lysosome localization” effect, indicating the steroid structures do not remarkably influence the intracellular localization behaviors of the nanoplexes. All in all, the results demonstrated the physico-chemical properties, cytotoxicity and gene transfection features could be tuned by appropriately control of steroid structures and hydrophobic/hydrophilic ratios of the steroid-based cationic polymers.

## Conflicts of interest

There are no conflicts to declare.

## Supplementary Material

RA-011-D1RA00223F-s001

## References

[cit1] Muhammad K., Zhao J., Gao B., Feng Y. (2020). Polymeric nano-carriers for on-demand delivery of genes via specific responses to stimuli. J. Mater. Chem. B.

[cit2] Keles E., Song Y., Du D., Dong W. J., Lin Y. (2016). Recent progress in nanomaterials for gene delivery applications. Biomater. Sci..

[cit3] Wang Y., Ye M., Xie R., Gong S. (2019). Enhancing the *in vitro* and *in vivo* stabilities of polymeric nucleic acid delivery nanosystems. Bioconjugate Chem..

[cit4] Jin L., Zeng X., Liu M., Deng Y., He N. (2014). Current progress in gene delivery technology based on chemical methods and nano-carriers. Theranostics.

[cit5] Suk J. S., Xu Q., Kim N., Hanes J., Ensign L. M. (2016). PEGylation as a strategy for improving nanoparticle-based drug and gene delivery. Adv. Drug Delivery Rev..

[cit6] Santo D., Mendonça P. V., Lima M. S., Cordeiro R. A., Cabanas L., Serra A., Coelho J. F. J., Faneca H. (2019). Poly(ethylene glycol)-block-poly(2-aminoethyl methacrylate hydrochloride)-based polyplexes as serum-tolerant nanosystems for enhanced gene delivery. Mol. Pharm..

[cit7] Van Bruggen C., Hexum J. K., Tan Z., Dalal R. J., Reineke T. M. (2019). Nonviral gene delivery with cationic glycopolymers. Acc. Chem. Res..

[cit8] Sun J., Sheng R., Luo T., Wang Z., Li H., Cao A. (2016). Synthesis of diblock/statistical cationic glycopolymers with pendant galactose and lysine moieties: gene delivery application and intracellular behaviors. J. Mater. Chem. B.

[cit9] Dai F., Liu W. (2011). Enhanced gene transfection and serum stability of polyplexes by PDMAEMA-polysulfobetaine diblock copolymers. Biomaterials.

[cit10] Yang J., Zhang Q., Chang H., Cheng Y. (2015). Surface-engineered dendrimers in gene delivery. Chem. Rev..

[cit11] Ong Z. Y., Yang C., Cheng W., Voo Z. X., Chin W., Hedrick J. L., Yang Y. Y. (2017). Biodegradable cationic poly(carbonates): effect of varying side chain hydrophobicity on key aspects of gene transfection. Acta Biomater..

[cit12] Sun Y., Xian L., Yu J., Yang T., Zhang J., Yang Z., Jiang J., Cai C., Zhao X., Yang L. (2017). *et al.*, Structure-function correlations of poly(amido amine)s for gene delivery. Macromol. Biosci..

[cit13] Bishop C. J., Kozielski K. L., Green J. J. (2015). Exploring the role of polymer structure on intracellular nucleic acid delivery via polymeric nanoparticles. J. Controlled Release.

[cit14] Zhang Y., Zhou Z., Chen M. (2018). The length of hydrophobic chain in amphiphilic polypeptides regulates the efficiency of gene delivery. Polymers.

[cit15] Liu Z., Zhang Z., Zhou C., Jiao Y. (2010). Hydrophobic modifications of cationic polymers for gene delivery. Prog. Polym. Sci..

[cit16] Lin D., Jiang Q., Cheng Q., Huang Y., Huang P., Han S., Guo S., Liang Z., Dong A. (2013). Polycation-detachable nanoparticles self-assembled from MPEG-PCL-g-SS-PDMAEMA for in vitro and in vivo siRNA delivery. Acta Biomater..

[cit17] Tang S., Huang Z., Zhang H., Wang Y., Hu Q., Jiang H. (2014). Design and formulation of trimethylated chitosan-graft-poly(ε-caprolactone) nanoparticles used for gene delivery. Carbohydr. Polym..

[cit18] Guo S., Huang Y., Wei T., Zhang W., Wang W., Lin D., Zhang X., Kumar A., Du Q., Xing J. (2011). Amphiphilic and biodegradable methoxy polyethylene glycol-block-(polycaprolactone-graft-poly(2-(dimethylamino)ethyl methacrylate)) as an effective gene carrier. Biomaterials.

[cit19] Han S., Cheng Q., Wu Y., Zhou J., Long X., Wei T., Huang Y., Zheng S., Zhang J., Deng L. (2015). *et al.*, Effects of hydrophobic core components in amphiphilic PDMAEMA nanoparticles on siRNA delivery. Biomaterials.

[cit20] Huang Y., Lin D., Jiang Q., Zhang W., Guo S., Xiao P., Zheng S., Wang X., Chen H., Zhang H.-Y. (2012). *et al.*, Binary and ternary complexes based on polycaprolactone-graft-poly(N,N-dimethylaminoethyl methacrylate) for targeted siRNA delivery. Biomaterials.

[cit21] Alhoranta A. M., Lehtinen J. K., Urtti A. O., Butcher S. J., Aseyev V. O., Tenhu H. J. (2011). Cationic amphiphilic star and linear block copolymers: synthesis, self-assembly, and in vitro gene transfection. Biomacromolecules.

[cit22] Eltoukhy A. A., Sahay G., Cunningham J. M., Anderson D. G. (2014). Niemann-Pick C1 affects the gene delivery efficacy of degradable polymeric nanoparticles. ACS Nano.

[cit23] Tagalakis A. D., Lee D. H. D., Bienemann A. S., Zhou H., Munye M. M., Saraiva L., McCarthy D., Du Z., Vink C. A., Maeshima R. (2014). *et al.*, Multifunctional, self-assembling anionic peptide-lipid nanocomplexes for targeted siRNA delivery. Biomaterials.

[cit24] Wang X., He Y., Wu J., Gao C., Xu Y. (2010). Synthesis and evaluation of phenylalanine-modified hyperbranched poly(amido amine)s as promising gene carriers. Biomacromolecules.

[cit25] Kim H. A., Park J. H., Lee S., Choi J. S., Rhim T., Lee M. (2011). Combined delivery of dexamethasone and plasmid DNA in an animal model of LPS-induced acute lung injury. J. Controlled Release.

[cit26] Piao C., Park J. H., Lee M. (2017). Anti-inflammatory therapeutic effect of adiponectin gene delivery using a polymeric carrier in an acute lung injury model. Pharm. Res..

[cit27] Cheng Q., Du L., Meng L., Han S., Wei T., Wang X., Wu Y., Song X., Zhou J., Zheng S. (2016). *et al.*, The promising nanocarrier for doxorubicin and siRNA Co-delivery by PDMAEMA-based amphiphilic nanomicelles. ACS Appl. Mater. Interfaces.

[cit28] Du L., Wang C., Meng L., Cheng Q., Zhou J., Wang X., Zhao D., Zhang J., Deng L., Liang Z. (2018). *et al.*, The study of relationships between pKa value and siRNA delivery efficiency based on Tri-block copolymers. Biomaterials.

[cit29] Guo S., Huang Y., Zhang W., Wang W., Wei T., Lin D., Xing J., Deng L., Du Q., Liang Z. (2011). *et al.*, Ternary complexes of amphiphilic polycaprolactone-graft-poly(N,N-dimethylaminoethyl methacrylate), DNA and polyglutamic acid-graft-poly(ethylene glycol) for gene delivery. Biomaterials.

[cit30] Lin D., Huang Y., Jiang Q., Zhang W., Yue X., Guo S., Xiao P., Du Q., Xing J., Deng L. (2011). *et al.*, Structural contributions of blocked or grafted poly(2-dimethylaminoethyl methacrylate) on PEGylated polycaprolactone nanoparticles in siRNA delivery. Biomaterials.

[cit31] Yue X., Zhang W., Xing J., Zhang B., Deng L., Guo S., Yang J., Zhang Q., Dong A. (2012). Self-assembled cationic triblock copolymer MPEG-b-PDLLA-b-PDMA nanoparticles as nonviral gene vector. Soft Matter.

[cit32] Sun J., Luo T., Sheng R., Li H., Chen S., Hu F., Cao A. (2013). Preparation of functional water-soluble low-cytotoxic poly(methacrylate)s with pendant cationic l-lysines for efficient gene delivery. Macromol. Biosci..

[cit33] Sun J., Luo T., Sheng R., Li H., Wang Z., Cao A. (2016). Intracellular plasmid DNA delivery by self-assembled nanoparticles of amphiphilic PHML-b-PLLA-b-PHML copolymers and the endocytosis pathway analysis. J. Biomater. Appl..

[cit34] Furgeson D. Y., Chan W. S., Yockman J. W., Kim S. W. (2003). Modified linear Polyethylenimine–cholesterol conjugates for DNA complexation. Bioconjugate Chem..

[cit35] Navarro G., Essex S., Sawant R. R., Biswas S., Nagesha D., Sridhar S., de ILarduya C. T., Torchilin V. P. (2014). Phospholipid-modified polyethylenimine-based nanopreparations for siRNA-mediated gene silencing: implications for transfection and the role of lipid components. Nanomedicine.

[cit36] Essex S., Navarro G., Sabhachandani P., Chordia A., Trivedi M., Movassaghian S., Torchilin V. P. (2015). Phospholipid-modified PEI-based nanocarriers for in vivo siRNA therapeutics against multidrug-resistant tumors. Gene Ther..

[cit37] Liu J., Feng M., Liang D., Yang J., Tang X. (2016). Vitamin E-labeled polyethylenimine for in vitro and in vivo gene delivery. Biomacromolecules.

[cit38] Eliyahu H., Makovitzki A., Azzam T., Zlotkin A., Joseph A., Gazit D., Barenholz Y., Domb A. J. (2005). Novel dextran–spermine conjugates as transfecting agents: comparing water-soluble and micellar polymers. Gene Ther..

[cit39] Neamnark A., Suwantong O., Bahadur R. K. C., Hsu C. Y. M., Supaphol P., Uludağ H. (2009). Aliphatic lipid substitution on 2 KDa polyethylenimine improves plasmid delivery and transgene expression. Mol. Pharm..

[cit40] Ercole F., Whittaker M. R., Quinn J. F., Davis T. P. (2015). Cholesterol modified self-assemblies and their application to nanomedicine. Biomacromolecules.

[cit41] Bajaj A., Kondaiah P., Bhattacharya S. (2008). Synthesis and gene transfection efficacies of PEI–Cholesterol-based lipopolymers. Bioconjugate Chem..

[cit42] Sevimli S., Sagnella S., Kavallaris M., Bulmus V., Davis T. P. (2012). Synthesis, self-assembly and stimuli responsive properties of cholesterol conjugated polymers. Polym. Chem..

[cit43] Bishop C. J., Kozielski K. L., Green J. J. (2015). Exploring the
role of polymer structure on intracellular nucleic acid delivery via polymeric nanoparticles. J. Controlled Release.

[cit44] Laskar P., Somani S., Altwaijry N., Mullin M., Bowering D., Warzecha M., Keating P., Tate R. J., Leung H. Y., Dufès C. (2018). Redox-sensitive, cholesterol-bearing PEGylated poly(propylene Imine)-based dendrimersomes for drug and gene delivery to cancer cells. Nanoscale.

[cit45] Kim G., Piao C., Oh J., Lee M. (2018). Self-assembled polymeric micelles for combined delivery of anti-inflammatory gene and drug to the lungs by inhalation. Nanoscale.

[cit46] Chen C. J., Wang J. C., Zhao E. Y., Gao L. Y., Feng Q., Liu X. Y., Zhao Z. X., Ma X. F., Hou W. J., Zhang L. R. (2013). *et al.*, Self-assembly cationic nanoparticles based on cholesterol-grafted bioreducible poly(amidoamine) for siRNA delivery. Biomaterials.

[cit47] Sheng R., Luo T., Zhu Y., Li H., Sun J., Chen S., Sun W., Cao A. (2011). The intracellular plasmid DNA localization of cationic reducible cholesterol-disulfide lipids. Biomaterials.

[cit48] Sheng R., Wang Z., Luo T., Cao A., Sun J., Kinsella J. M. (2018). Skeleton-controlled pDNA delivery of renewable steroid-based cationic lipids, the endocytosis pathway analysis and intracellular localization. Int. J. Mol. Sci..

[cit49] Gibson T. J., Smyth P., Semsarilar M., McCann A. P., McDaid W. J., Johnston M. C., Scott C. J., Themistou E. (2020). Star polymers with acid-labile diacetal-based cores synthesized by aqueous RAFT polymerization for intracellular DNA delivery. Polym. Chem..

[cit50] Olden B. R., Cheng Y., Yu J. L., Pun S. H. (2018). Cationic polymers for non-viral gene delivery to human T cells. J. Controlled Release.

[cit51] Agarwal S., Zhang Y., Maji S., Greiner A. (2012). PDMAEMA-based gene delivery materials. Mater. Today.

[cit52] Skey J., O'Reilly R. K. (2008). Facile one pot synthesis of a range of reversible addition-fragmentation chain transfer (RAFT) agents. Chem. Commun..

