# Expression of concern: Natural steroid-based cationic copolymers cholesterol/diosgenin-*r*-PDMAEMAs and their pDNA nanoplexes: impact of steroid structures and hydrophobic/hydrophilic ratios on pDNA delivery

**DOI:** 10.1039/d3ra90019c

**Published:** 2023-03-15

**Authors:** Zhao Wang, Jingjing Sun, Mingrui Li, Ting Luo, Yulin Shen, Amin Cao, Ruilong Sheng

**Affiliations:** a Department of Radiology, Shanghai Tenth People's Hospital, School of Medicine, Tongji University Shanghai 200072 China; b School of Material Engineering, Jinling Institute of Technology Nanjing 211169 China; c CAS Key Laboratory of Synthetic and Self-assembly Chemistry for Organic Functional Molecules, Shanghai Institute of Organic Chemistry, Chinese Academy of Sciences 345 Lingling Road Shanghai 200032 China; d CQM-Centro de Quimica da Madeira, Universidade da Madeira, Campus da Penteada Funchal Madeira 9000-390 Portugal ruilong.sheng@staff.uma.pt

## Abstract

Expression of concern for ‘Natural steroid-based cationic copolymers cholesterol/diosgenin-*r*-PDMAEMAs and their pDNA nanoplexes: impact of steroid structures and hydrophobic/hydrophilic ratios on pDNA delivery’ by Zhao Wang *et al.*, *RSC Adv.*, 2021, **11**, 19450–19460, DOI: https://doi.org/10.1039/D1RA00223F.

*RSC Advances* is publishing this expression of concern in order to alert our readers that we are presently unsure of the reliability of the data reported in Fig. 2 of the article. Fig. 2a and b are identical. The authors have requested Fig. 2b be removed from the article but are unable to provide a replacement for Fig. 2b. An independent expert has stated it would not be appropriate to remove Fig. 2b completely and requested the authors reproduce their results.

An expression of concern will continue to be associated with the article until we receive conclusive evidence regarding the reliability of the reported data.

Laura Fisher

9th March 2023

Executive editor, *RSC Advances*

## Supplementary Material

