# Correction and removal of expression of concern: Natural steroid-based cationic copolymers cholesterol/diosgenin-*r*-PDMAEMAs and their pDNA nanoplexes: impact of steroid structures and hydrophobic/hydrophilic ratios on pDNA delivery

**DOI:** 10.1039/d3ra90073h

**Published:** 2023-08-04

**Authors:** Zhao Wang, Jingjing Sun, Mingrui Li, Ting Luo, Yulin Shen, Amin Cao, Ruilong Sheng

**Affiliations:** a Department of Radiology, Shanghai Tenth People's Hospital, School of Medicine, Tongji University Shanghai 200072 China; b School of Material Engineering, Jinling Institute of Technology Nanjing 211169 China; c CAS Key Laboratory of Synthetic and Self-assembly Chemistry for Organic Functional Molecules, Shanghai Institute of Organic Chemistry, Chinese Academy of Sciences 345 Lingling Road Shanghai 200032 China; d CQM-Centro de Quimica da Madeira, Universidade da Madeira, Campus da Penteada Funchal Madeira 9000-390 Portugal ruilong.sheng@staff.uma.pt

## Abstract

Correction and removal of expression of concern for ‘Natural steroid-based cationic copolymers cholesterol/diosgenin-*r*-PDMAEMAs and their pDNA nanoplexes: impact of steroid structures and hydrophobic/hydrophilic ratios on pDNA delivery’ by Zhao Wang *et al.*, *RSC Adv.*, 2021, **11**, 19450–19460, https://doi.org/10.1039/D1RA00223F.

Fig. 2a and b in the original article are identical. The authors have now provided replacement data for Fig. 2b, shown here. There was an error in the original caption and the caption has been updated to state ‘Agarose gel retardation assay’.

**Fig. 2:**
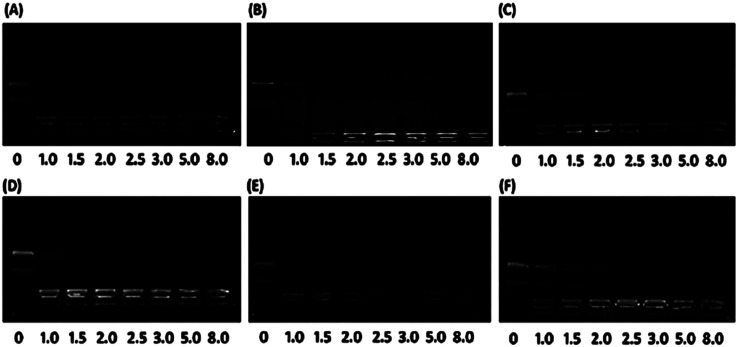
Agarose gel retardation assay of plasmid DNA affinity under various N/P charge ratios (0, 1.0, 1.5, 2.0, 2.5, 3.0, 5.0, 8.0) for the (A) Chol-P1/pDNA; (B) Chol-P2/pDNA; (C) Chol-P3/pDNA; (D) Dios-P1/pDNA; (E) Dios-P2/pDNA; (F) Dios-P3/pDNA.

An independent expert has viewed the corrected data for Fig. 2b and has concluded that it is consistent with the discussions and conclusions presented.

This correction supersedes the information provided in the Expression of Concern related to this article.

The authors regret additional errors in the manuscript.

In Table 1 the units for *M*_n,NMR_ and *M*_n,GPC_ should be ×10 kg mol^−1^.

On page 19451, in the left column, in paragraph 2, line 9 and in the right column, in paragraph 3, line 10 the text ‘(dimethylamino)ethyl methacrylate (DMAEMA)’ should have been written as ‘2-(dimethylamino)ethyl methacrylate (DMAEMA)’.

The authors and the Royal Society of Chemistry apologise for these errors and any consequent inconvenience to authors and readers.

## Supplementary Material

