# Supplementary material for: Natural steroid-based cationic copolymers cholesterol/diosgenin-r-PDMAEMAs and their pDNA nanoplexes: impact of steroid structures and hydrophobic/hydrophilic ratios on pDNA delivery
Source: RSC Adv. 2021 Jun 1;11(32):19450–60. doi: 10.1039/d1ra00223f (PMC9033666; doi:10.1039/d1ra00223f)
Supplement: RA-011-D1RA00223F-s001 [file RA-011-D1RA00223F-s001.pdf]

Electronic Supplementary Material (ESI) for RSC Advances.
This journal is © The Royal Society of Chemistry 2021

Supplementary Information

Natural Steroid-based Cationic Copolymers Cholesterol/Diosgenin-*r*-PDMAEMAs and their pDNA Nanoplexes: Impact of Steroid Structures and Hydrophobic/Hydrophilic Ratios on pDNA delivery

Zhao Wang[1,2,3], Jingjing Sun[3], Mingrui Li[3], Ting Luo[3], Yulin Shen[2], Amin Cao[3]*, Ruilong Sheng[1,3,4]*

1. *Department of Radiology, Shanghai Tenth People's Hospital, School of Medicine, Tongji University, Shanghai 200072, China.*
2. *School of Material Engineering, Jinling Institute of Technology, Nanjing, 211169, China.*
3. *CAS Key Laboratory of Synthetic and Self-assembly Chemistry for Organic Functional Molecules, Shanghai Institute of Organic Chemistry, Chinese Academy of Sciences, 345 Lingling Road, Shanghai 200032, China*
4. *CQM-Centro de Quimica da Madeira, Universidade da Madeira, Campus da Penteada, 9000-390, Funchal, Madeira, Portugal. E-mail: ruilong.sheng@staff.uma.pt (R. Sheng).*

Contents

S1. Synthesis and characterization of the cholesterol-based 6-(Cholesteryloxy)hexyl methacrylate (MA6chol) and diosgenin-based 6-(Diosgeninyloxy)hexyl methacrylate (MA6Dios) monomers.

Scheme S1a. Synthetic routes of the 6-(Cholesteryloxy)hexyl methacrylate (MA6chol) monomer, **Figure S1a.** ^{1}H NMR spectra of the 6-(Cholesteryloxy)hexyl methacrylate monomer (MA6Chol) monomer;

Scheme S1b. Synthetic routes of the 6-(Diosgeninyloxy)hexyl methacrylate (MA6Dios) monomer, **Figure S1b.** ^{1}H NMR spectra of the 6-(Diosgeninyloxy)hexyl methacrylate (MA6Dios) monomer

S2. Synthesis of the PMA6Chol-*r*-PDMAEMA and PMA6Dios-*r*-PDMAEMA cationic random copolymers and related RAFT polymerization mechanisms

S1. Synthesis and characterization of the cholesterol-based 6-(Cholesteryloxy)hexyl methacrylate (MA6chol) and diosgenin-based 6-(Diosgeninyloxy)hexyl methacrylate (MA6Dios) monomers.[1]

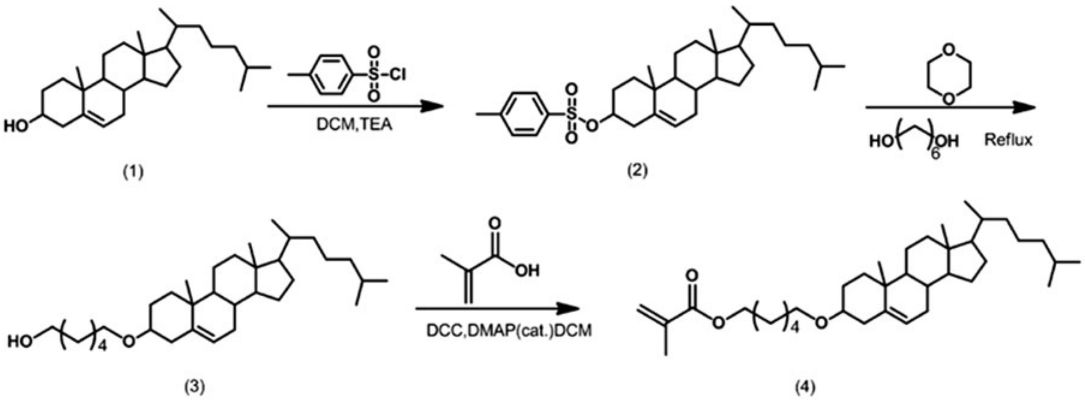

Scheme S1a. Synthetic routes of the 6-(Cholesteryloxy)hexyl methacrylate (MA6chol) monomer

Synthesis of cholesteryl tosylate (2)

In a 500 mL flask, cholesterol (1) (38.7 g，0.1 mol) and p-Tosyl chloride（TsCl, 38.1 g, 0.2 mol）were dissolved in 300 mL CH_2Cl_2 and 30 mL triethylamine, the reaction mixture was stirred at ambient temperature for 24 h, then the solvent was removed under reduced pressure, and the residue was gently poured into 5% K_2CO_3 solution, the crude product was washed with distilled water for several times, dissolved in CH_2Cl_2, dried with anhydrous $MgSO_4$, filtrated and concentrated, then recrystallized with acetone to obtain cholesteryl tosylate (2) as white solid. (54.5g, Yield: 92%).

^{1}H NMR (300MHz, $CDCl_3$, δ in ppm): 7.81-7.79 (2H, d, *J*= 7.5 Hz, o-Ar*H*), 7.35-7.33 (2H, d, *J*= 7.7 Hz, m-Ar*H*), 5.30 (1H, s, =C*H,* cholesterol), 4.36-4.29 (1H, m, SO_2OCH), 2.46 (3H, s, Ar-CH_3), 2.30-0.65 (43H, m, other protons on cholesterol and alkyl chain)

Synthesis of 6-Cholesteryloxyhexanol (3)

Cholesteryl tosylate (2) (8.7 g, 0.015 mol) and 1,6-hexanediol (18 g, 0.15 mol) dissolved in 100 mL 1,4-dioxane and refluxed at 100°C for 16 h, the organic solvent was removed under reduced pressure and the brown residue was dissolved with 200 mL CH_2Cl_2, washed by saturated $NaHCO_3$, distilled water and saturated NaCl (each at 100 mL), dried with anhydrous $MgSO_4$, filtrated and concentrated, then purified with column chromatography (silica gel, n-hexane/ethyl acetate, v/v=3/1), 6-Cholesteryloxyhexanol (3) was obtained as white solid. (3.94 g, Yield: 54%).

^1H NMR (300MHz, $CDCl_3$, δ in ppm): 5.34 (1H, s, =C*H,* cholesterol), 3.65 (2H, t, *J*= 5.1 Hz, (HO)CH_2)), 3.46 (2H, t, *J*= 5.2 Hz, (CHO)CH_2), 3.19-3.11 (1H, m, OC*H*), 2.39-0.65 (52H, m, other protons on cholesterol and alkyl chain)

Synthesis of 6-(Cholesteryloxy)hexyl methacrylate（MA6Chol）monomer (4)[1]

6-Cholesteryloxyhexanol (3)(11.5 g, 0.024 mol), DCC (12.4 g, 0.06 mol) and DMAP (1.47 g, 0.012 mol) dissolved in dried 200 mL anhydrous CH_2Cl_2, methylacylated (5.2 g, 0.06 mol) was added dropwise, the reaction mixture was stirred at ambient temperature for 24 h, which was filtrated, concentrated and then purified with column chromatography (silica gel, n-hexane/ethyl acetate, v/v=2/1) to afford 6-(Cholesteryloxy)hexyl methacrylate (MA6Chol) monomer (4) as white solid (10.6 g，Yield: 82%).

^1H NMR (300MHz, $CDCl_3$, δ in ppm): 6.10 (m, 1H, =CH*H*), 5.55 (m, 1H, =C*H*H), 5.34 (s, 1H, =C*H*R, cholesterol), 4.14 (t, 2H, *J*= 5.0 Hz, COOCH_2), 3.45 (t, 2H, *J*= 5.2 Hz, CH_2OR), 3.12 (m, 1H, OC*H*R), 1.94 (s, 3H,=CCH_3), 2.38-0.67 (m, 54H, other protons on cholesterol and alkyl chain)

^{13}C NMR (75MHz, $CDCl_3$, δ in ppm):167.5, 141.1, 136.5, 125.2, 121.4, 79.0, 76.7, 67.9, 64.7, 56.7, 56.1, 50.2, 42.3, 39.8, 39.5, 39.2, 37.3, 36.9, 36.2, 35.8, 31.9, 30.1, 28.6, 28.4, 28.2, 28.0, 25.9, 24.3, 23.8, 22.5, 21.0, 19.4, 18.7, 18.3, 11.8

FTIR (in cm^{-1}): 2932 ($\nu_{C\text{-}H}$), 2856 ($\nu_{=C\text{-}H}$), 1716 ($\nu_{C=O}$), 1638 ($\nu_{C=C}$), 1467 ($\nu_{C\text{-}C}$), 1365 ($\delta_{C\text{-}H}$), 1298($\delta_{C\text{-}O}$), 1169($\delta_{C\text{-}O\text{-}C}$), 1102, 941

ESI-MS [M+Na^+] (in m/z): 577.4 (Calculated 577.5)

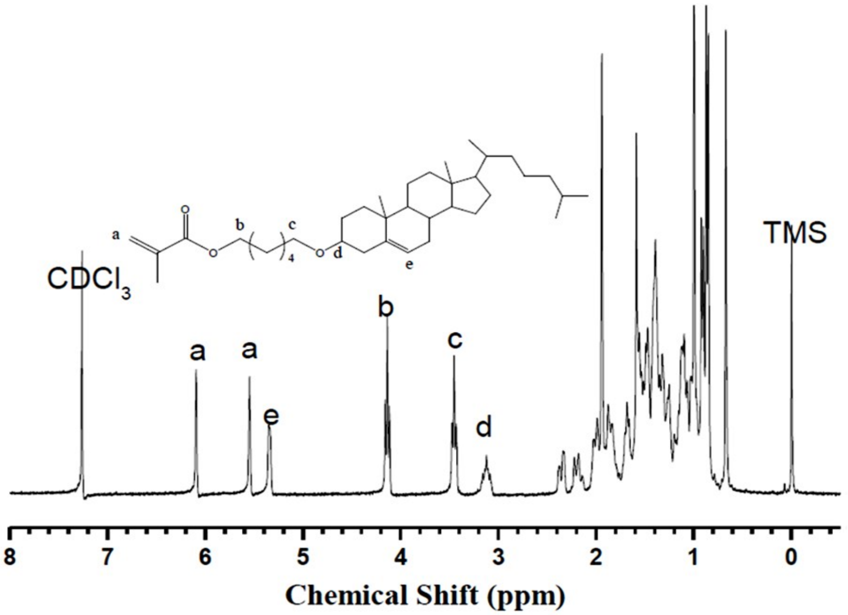

Figure S1a. ^1H NMR spectra of the 6-(Cholesteryloxy)hexyl methacrylate (MA6Chol) monomer

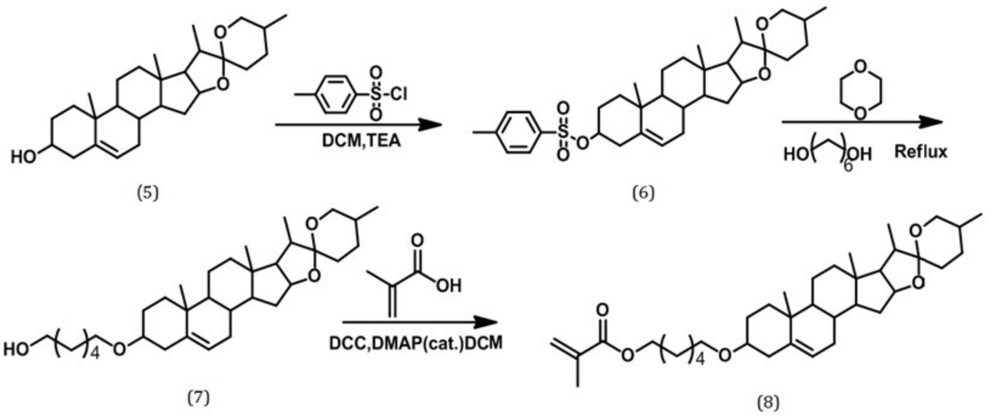

Scheme S1b. Synthetic routes of the 6-(Diosgeninyloxy)hexyl methacrylate (MA6Dios) monomer

Synthesis of diosgeninyl tosylate (6)

In a 500 mL flask, diosgenin (5) (10.0 g，0.024 mol), p-Tosyl chloride（TsCl, 9.0 g, 0.047 mol）and DMAP (3.69 g，30 mmol) dissolved in 150 mL CH_2Cl_2, the reaction mixture was stirred at ambient temperature for 24 h, then the solvent was removed under reduced pressure, and the residue was gently poured into 5% K_2CO_3 solution, the crude product was washed with distilled water for several times, dissolved in CH_2Cl_2, dried with anhydrous $MgSO_4$, filtrated and concentrated, then recrystalled with acetone, diosgeninyl tosylate (6) was obtained as white solid. (13.0 g，Yield: 95%).

^{1}H NMR (300MHz, $CDCl_3$, δ in ppm): 7.83-7.76 (2H, d, *J*= 7.4 Hz, o-Ar*H*), 7.36-7.33 (2H, d, *J*= 7.5 Hz, m-Ar*H*), 5.34 (1H, s, =C*H,* diosgenin), 4.36-4.28 (1H, m, SO_2OC*H*), 2.45 (3H, s, Ar-CH_3), 2.34-0.63 (44H, m, other protons on diosgenin and alkyl chain)

Synthesis of 6-Diosgeninyloxyhexanol (7)

Diosgeninyl tosylate (6) (8.5 g, 0.015 mol) and 1,6-hexanediol (18 g, 0.15 mol) dissolved in 100 mL 1,4-dioxane and refluxed at 100°C for 16 h, the organic solvent was removed under reduced pressure and the brown residue was dissolved with 200 mL CH_2Cl_2, washed by saturated $NaHCO_3$, water and saturated NaCl solution (each at 100 mL), dried with anhydrous $MgSO_4$, filtrated and concentrated, then purified with column chromatography (silica gel, hexane/ethyl acetate, v/v=3/1), 6-Diosgeninyloxyhexanol (7) was obtained as white solid. (3.62 g, Yield: 51%).

^{1}H NMR (300MHz, $CDCl_3$, δ in ppm): 5.32 (1H, s, =C*H,* diosgenin), 3.62 (2H, t, *J*= 5.0 Hz, (HO)CH_2)), 3.47 (2H, t, *J*= 5.0 Hz, (CHO)CH_2), 3.20-3.10 (1H, m, OC*H*), 2.34-0.63 (52H, m, other protons on diosgenin and alkyl chain).

Synthesis of 6-(Diosgeninyloxy)hexyl methacrylate monomer (MA6Dios) (8)

In a 250 mL flask, 6-Diosgeninyloxyhexanol (7) (11.3 g, 0.024 mol), DCC (12.4 g, 0.06 mol) and DMAP (1.47 g, 0.012 mol) dissolved in dried 200 mL anhydrous CH_2Cl_2, methylacylated (5.2 g, 0.06 mol) was added dropwise, the reaction mixture was stirred at ambient temperature for 24 h, filtrated and concentrated，then purified with column chromatography (silica gel, hexane/ethyl acetate, v/v=2/1) to afford 6-(Cholesteryloxy)hexyl methacrylate (MA6Chol) monomer (4) as white solid (9.3 g, Yield: 72%).

^{1}H NMR (300MHz, $CDCl_3$, δ in ppm): 6.12 (m, 1H, =CH*H*), 5.55 (m, 1H, =C*H*H), 5.34 (s, 1H,=C*H*R, diosgenin), 4.15 (t, 2H, *J*= 5.2 Hz, COOCH_2), 3.45 (t, 2H, *J*= 5.4 Hz, CH_2OR), 3.12 (m, 1H, OC*H*R), 1.94 (s, 3H,=CCH_3), 2.43-0.67 (m, 55H, other protons on diosgenin and alkyl chain)

^{13}C NMR (75MHz, $CDCl_3$, δ in ppm):167.5, 141.1, 136.4, 125.6, 121.3, 109.6, 80.9, 78.6, 67.9, 65.8, 62.7, 56.6, 50.8, 42.0, 40.1, 39.8, 39.5, 37.5, 37.2, 32.8, 31.9, 31.3, 30.1, 29.6, 29.0, 25.8, 25.6, 21.1, 19.7, 17.8, 17.1, 15.5, 14.8,14.7

ESI-MS [M+Na$^+$] (in m/z): 605.4 (Calculated 605.5)

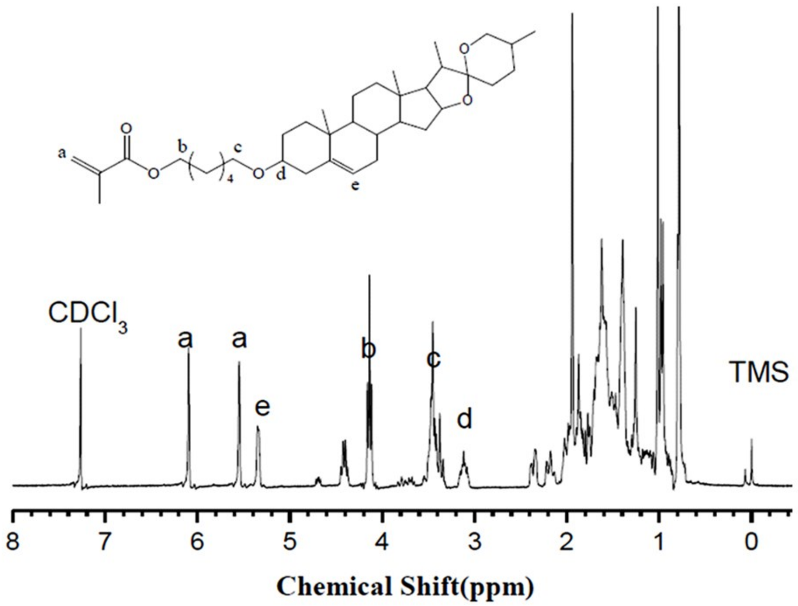

Figure S1b. ^{1}H NMR spectra of the 6-(Diosgeninyloxy)hexyl methacrylate (MA6Dios) monomer

S2. Synthesis of the PMA6Chol-*r*-PDMAEMA and PMA6Dios-*r*-PDMAEMA cationic random copolymers and related RAFT polymerization mechanisms

Predetermined amount of the as-synthesized MA6Chol/MA6Dios monomers, RAFT chain transfer agent DDMAT and initiator AIBN (as shown in **Table S2**) were dissolved in freshly distilled toluene and added into a dried Schlenk tube with a magnetic stirrer. The reaction mixture was deoxygenated with freeze-pump-thawing for three times and immersed into an oil-bath thermostated at 80℃ for 12 h under nitrogen atmosphere. The reaction was stopped by cooling to 0℃ in an ice bath and exposed to the air, and added dropwise into cooled n-hexane to precipitate the resulting cationic polymers, which were further washed with excessive n-hexane and dried at 40℃ under vacuum overnight to give the final product of Steroid-*r*-PDMAEMA cationic polymers: PMA6Chol-*r*-PDMAEMA and PMA6Dios-*r*-PDMAEMA as light yellow powder.

Table S2. The feeding ratio of the reactants for preparation of the PMA6Chol-*r*-PDMAEMA and PMA6Dios-*r*-PDMAEMA cationic random copolymers

**Entry 1**	**MA6Chol/ mmol**	**DMAEMA/ mmol**	**DDMAT/mol**	**AIBN/mol**
**Chol-P1**	0.10	3.99	0.02	6.7×10^{-3}
**Chol-P2**	0.20	3.99	0.02	6.7×10^{-3}
**Chol-P3**	0.44	3.99	0.02	6.7×10^{-3}
**Entry 2**	**MA6Dios/ mmol**	**DMAEMA/ mmol**	**DDMAT/mol**	**AIBN/mol**
**Dios-P1**	0.12	3.99	0.02	6.7×10^{-3}
**Dios-P2**	0.24	3.99	0.02	6.7×10^{-3}
**Dios-P3**	0.54	3.99	0.02	6.7×10^{-3}

The related RAFT polymerization mechanisms were presented as below:

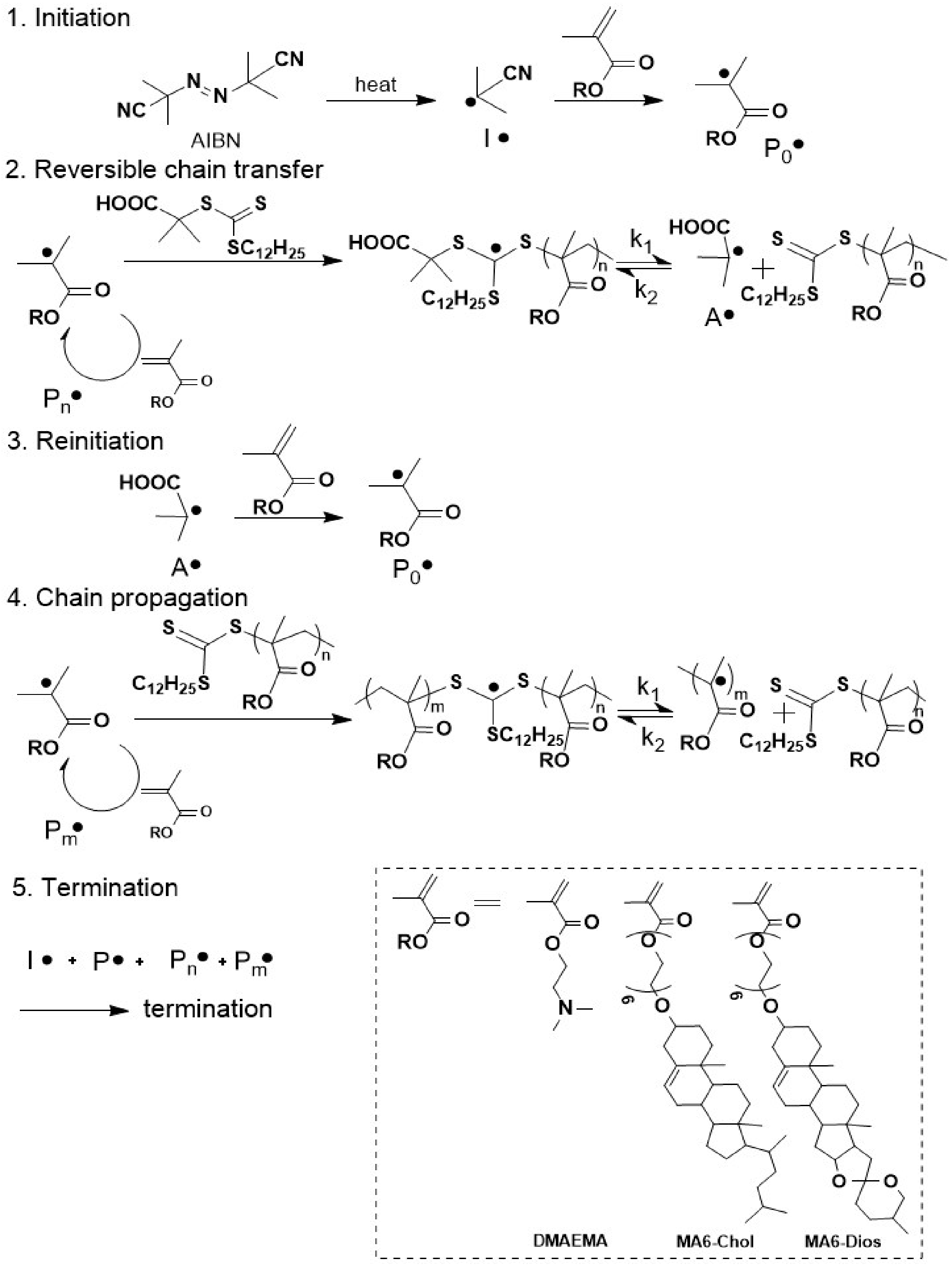

References:

[1]. a) Zhao Wang, Ting Luo, Ruilong Sheng, Hui Li, Jingjing Sun, and Amin Cao. *Biomacromolecules* **2016**, *17*, 98-110; b) Shengdian Chen, Fangzhen Hu, Ruilong Sheng, Amin Cao. *Acta Polymerica Sinica* **2013**, *13*,102-111.
